# Incidence and relative risk of hemorrhagic events associated with ramucirumab in cancer patients: a systematic review and meta-analysis

**DOI:** 10.18632/oncotarget.11097

**Published:** 2016-08-05

**Authors:** Rui Tian, Hong Yan, Fei Zhang, Peng Sun, Xucai Zheng, Yi Zhu, Qing Wang, Jie He

**Affiliations:** ^1^ Department of Pathology, Anhui Cancer Hospital, Hefei, Anhui, China; ^2^ Collaborative Innovation Center for Cancer Medicine, Guangzhou, Guangdong, China; ^3^ State Key Laboratory of Oncology in South China, Guangzhou, Guangdong, China; ^4^ Department of Medical Oncology, Sun Yat-sen University Cancer Center, Guangzhou, Guangdong, China; ^5^ Department of Head, Neck and Breast Surgery, Anhui Cancer Hospital, Hefei, Anhui, China; ^6^ Department of Gynaecology and Obstetrics, Anhui Provincial Hospital, Hefei, Anhui, China

**Keywords:** ramucirumab, hemorrhagic events, VEGF, VEGFR, bleeding

## Abstract

The purpose of this study was to investigate the overall incidence and relative risk (RR) of hemorrhagic events in cancer patients treated with ramucirumab. 298 potentially relevant citations on ramucirumab from Pubmed, Web of Science and the Cochrane Database, as well as abstracts presented at conferences (all up to March 2016) were identified through our initial search. Only phase II and III prospective clinical trials of ramucirumab among cancer patients with toxicity records on hemorrhagic events were selected for final analysis. Data was extracted from the original studies by two independent reviewers. The overall incidence, RR, and 95% confidence intervals (CI) were calculated using fixed or random effects models according to the heterogeneity of the enrolled studies. The statistical analysis was performed by STATA version 11.0 (Stata Corporation, College Station, TX). 4963 patients with a variety of solid tumors from eleven eligible studies were selected into our analysis. The results demonstrated that the overall incidences of all-grade and high-grade hemorrhagic events in cancer patients were 27.6% (95% CI, 18.7-36.5%) and 2.3% (95% CI, 1.3-3.2%), respectively. The RR of hemorrhagic events of ramucirumab compared to control was significantly increased for low-grade (RR, 2.06; 95% CI, 1.85-2.29, *p* < 0.001), but not for high-grade (RR, 1.19, 95% CI, 0.80-1.76, *p*=0.39) hemorrhagic events. Hemorrhagic events associated with ramucirumab are modest and manageable while patients could continue to receive ramucizumab treatment to achieve their maximum clinical benefits.

## INTRODUCTION

Angiogenesis, the multistep formation of new capillaries and blood vessels, plays a pivotal role in tumor growth, invasion, and metastasis [1]. Results from preclinical and clinical studies have confirmed that both VEGF and VEGFR-2-mediated signalling and angiogenesis make great contributions to the pathogenesis of a variety of malignant tumors and provide potential therapeutic targets for antiangiogenic treatment [2]. Cancer patients who were treated with antiangiogenic inhibitors by blocking VEGF/VEGFR-2 pathway have obtained varying degrees clinical benefit. Anti-VEGF monoclonal antibody (bevacizumab), VEGF fusion protein (aflibercept), and VEGF tyrosine kinase inhibitors (sorafenib, sunitinib, vandetanib, pazopanib, etc) are now the most widely used antiangiogenic inhibitors in clinical practice.

Ramucirumab (IMC-1121B, ImClone Systems, Bridgewater, NJ, USA) is a fully human IgG1 monoclonal antibody that specifically binds to the VEGFR-2 extracellular domain with high affinity, preventing binding of all VEGF ligands and receptor activation [3]. Results from several phase III clinical trials among multiple tumor types have confirmed the efficacy of ramucirumab [4-8]. The inhibition of the VEGFR-2 receptor by ramucirumab in second-line treatment for advanced gastric cancer (GAC) or gastroesophageal junction adenocarcinoma (GEJC) improved survival when given alone or in combination with paclitaxel [5-6], and for non-small-cell lung cancer (NSCLC) when given in combination with docetaxel [7].

VEGF/VEGFR2 inhibitors have a unique series of adverse events, which are quite different from traditional cytotoxic agents. Similar to other antiangiogenic agents (bevacizumab, aflibercept, and multitargeted antiangiogenic tyrosine kinase inhibitors), hemorrhagic events (epistaxis, gastrointestinal hemorrhage/bleeding and pulmonary hemorrhage) are one kind of the major adverse events reported in clinical trials of ramucirumab. Hemorrhagic events may cause severe outcomes, some could be life-threatening, leading to the limited usage of ramucirumab, thus the recognition and management of hemorrhagic events in cancer patients who are receiving ramucirumab are seriously important issues [9]. However, the overall incidence and relative risk (RR) of hemorrhagic events with ramucirumab have yet to be defined. Therefore, we conducted a systematic review and meta-analysis of available clinical trials to determine the incidence and RR of hemorrhagic events in cancer patients treated with ramucirumab.

## MATERIALS AND METHODS

### Literature-search strategy

We systematically searched Pubmed, Web of Science and the Cochrane Database (up to March 2016) using various combinations of the terms: (“ramucirumab” or “IMC-1121B” or “LY3009806”), and (“cancer” or “carcinoma” or “tumor” or malignancy” or “neoplasia”) and (“clinical trial” or “prospective trials” or “randomized controlled trial”). Furthermore, the American Society of Clinical Oncology (ASCO) and European Society of Medical Oncology (ESMO) abstracts database of the annual meetings in the past ten years was also searched. The search was performed with restriction to English language, and references in the primary publications were also assessed to find additional studies. When multiple reports describing the same population were published, the most recent or complete one was selected for final analysis. All studies were selected and systemically reviewed in accordance with the Preferred Reporting Items for Systematic Reviews and Meta-Analyses (PRISMA) statement.

### Inclusion criteria

Relevant clinical trials that met the following criteria were included: (1) patients were pathologically diagnosed with solid tumors; (2) prospective phase II and III clinical trials; (3) patients assigned to receive ramucirumab at 8 mg/kg Q2W or 10 mg/kg Q3W; (4) clinical trials with toxicity profiles on hemorrhagic events, including epistaxis, eye hemorrhage, ecchymosis or petechiae, gastrointestinal bleeding/hemorrhage, injection-site hemorrhage, gum hemorrhage, hematemesis, hematuria, hemoptysis, nonspecific hemorrhage, hemothorax, melena, menorrhagia, metrorrhagia, purpura, rectal hemorrhage, retroperitoneal hemorrhage, central nerve system hemorrhage, vaginal hemorrhage and other related hemorrhagic events; (5) there was sufficient data for extraction. Review articles, irrelevant topics, non-comparative studies, case reports, and animal experimental studies were excluded.

### Data extraction and clinical end points

The publications and data were reviewed and extracted by two independent investigators (T.R. and Z.F.). The relevant information of each study including: (1) article or publication information, such as first author's name, year of publication, etc.; (2) patient characteristics, such as diagnosis, age, gender, etc.; (3) study designation information, such as phase, total sample size, sample size per arm; (4) information about treatment, such as treatment approach, dose and cycle of ramucirumab used; (5) hemorrhagic events from the safety and toxicity profile and so on were carefully extracted, and they were recorded to a data collection form and then entered into an electronic database.

And any discrepancy was resolved by consensus. Hemorrhagic events were assessed and recorded according to the National Cancer Institute's Common Toxicity Criteria for Adverse Events (NCI-CTCAE) (version 3.0 or 4.0), which has been widely used in cancer clinical trials. They define the grading of hemorrhagic events as follows: grade 1, mild bleeding (medical or invasive intervention not indicated); grade 2, moderate and symptomatic bleeding (medical and/or invasive intervention indicated); grade 3, in need of transfusion, interventional radiology, or operative intervention; grade 4, life-threatening consequences (major urgent intervention indicated); and grade 5, death.

### Statistical analysis

Statistical analysis of the overall incidence and RR for hemorrhagic events in cancer patients who received ramucirumab were performed using STATA version 11.0 (Stata Corporation, College Station, TX). We derived the proportion and calculated the 95% confidence interval (CI) of patients with all-grade and high-grade hemorrhagic events from each study. For randomized controlled studies, we also calculated and compared the RRs of low grade and high-grade hemorrhagic events. The χ^2^-based Q statistic was applied to determine the heterogeneity between selected studies. And the heterogeneity was considered to be statistically significant when *P*_heterogeneity_ < 0.10 or *I^2^ >* 50%. If heterogeneity existed, data was analyzed using a random-effects model, otherwise, a fixed-effects model was used. RR > 1 indicated more hemorrhagic events in ramucirumab containing treatment arm; and vice versa. We further investigated the differences in incidences and RRs of hemorrhagic events according to different tumor types and ramucirumab dosages. The Funnel plot was used to assess the potential publication bias regarding our primary endpoint (relative risk of hemorrhagic events). A two-sided *p* value of < 0.05 was considered statistically significant. And all CIs had a two-sided probability and with a coverage of 95%.

## RESULTS

### Evidence synthesis

**Figure 1:**
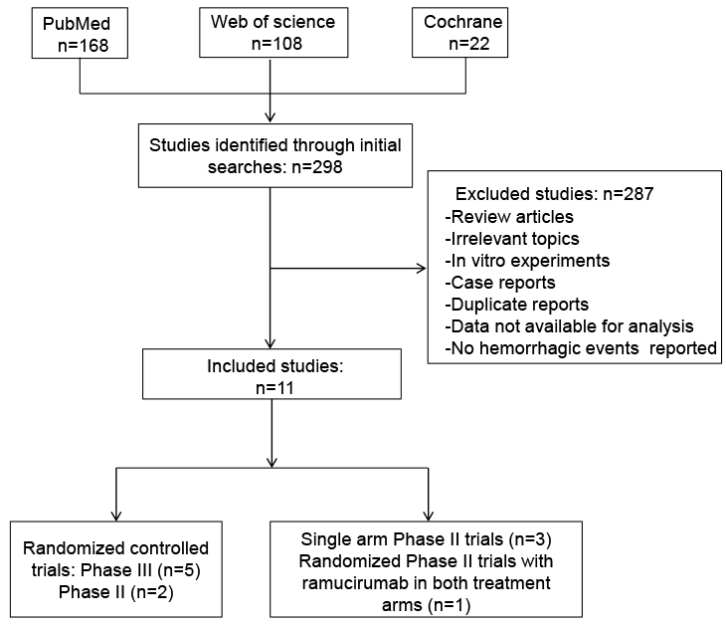
Flow diagram of studies identified, included, and excluded

A total of 298 potentially relevant studies on ramucirumab were identified through initial database searching. Eleven eligible studies met the inclusion criteria and were selected into the final analysis [4-8,10-15] (Figure 1). Examination of references in the primary publications did not yield any additional studies for evaluation. Review articles, irrelevant topics, non-comparative studies, case reports, and animal experimental studies were excluded.

### Characteristics of eligible studies

A total of 4963 patients from 11 enrolled studies were included for the purpose of analysis. Five of the selected trials were phase III studies [4-8], while the remaining six were phase II studies [10-15]. Seven of the trials were randomized controlled studies (RCTs) [4-8, 10, 11], whereas, the other four were non-randomized controlled studies (non-RCTs) [12-15], including one study evaluating ramucirumab in both treatment arms [12]. Patients were required to have an Eastern Cooperative Oncology Group (ECOG) performance status between 0 and 1, adequate haematological, hepatic and renal function. Underlying malignancies included previously treated advanced NSCLC (two studies) [7, 10], previously treated GAC or GEJC (two studies) [5, 6], metastatic breast cancer (MBC) (two studies) [8, 11], previously treated metastatic renal cell cancer (RCC) (one study) [13], previously untreated metastatic colorectal cancer (mCRC) (one study) [4], advanced hepatocellular cancer (HCC) (one study) [15], metastatic melanoma (one study) [12] and epithelial ovarian carcinoma (EOC) or primary peritoneal carcinoma (PPC) (one study) [14]. Their characteristics were listed in Table 1. All eleven studies had adequate data for data extraction.

**Table 1:** Baseline characteristics of studies included in the meta-analysis

Author(s)	Year	Phase	Underlying	Treatment regimen	No. of patients for analysis	No. of hemorrhagic events		Study quality
			malignancy			Low-grade	High-grade	
Tabernero *et al.*	2015	III	mCRC	ramucirumab 8 mg/kg Q2W + FOLFIRI	529	219	13	5
				vs placebo + FOLFIRI	528	111	9	
Wilke *et al.*	2014	III	GAC or GEJC	ramucirumab 8 mg/kg Q2W + PTX	327	123	14	5
				vs placebo + PTX	329	51	8	
Fuchs *et al.*	2014	III	GAC or GEJC	ramucirumab 8 mg/kg Q2W	236	30	8	5
				vs placebo	115	13	3	
Garon *et al.*	2014	III	NSCLC	ramucirumab 10 mg/kg Q3W + DOC	627	181	15	5
				vs placebo + DOC	618	94	14	
Mackey *et al.*	2014	III	MBC	ramucirumab 10 mg/kg Q3W + DOC	752	361	7	3
				vs placebo + DOC	382	85	7	
Doebele *et al.*	2015	II	NSCLC	ramucirumab 10 mg/kg Q3W + PEM + PLT	67	17	0	2
				vs PEM + PLT	69	5	0	
Yardley *et al.*	2014	II	MBC	ramucirumab 10 mg/kg Q3W + ERI	71	12	1	3
				vs ERI	70	3	0	
Carvajal *et al.*	2014	II	Melanoma	ramucirumab 10 mg/kg Q3W + DAC	52	7	1	-
				vs ramucirumab 10 mg/kg Q3W	50	4	0	
Garcia *et al*.	2014	II	RCC	ramucirumab 8 mg/kg Q2W	39	12	0	-
Penson *et al.*	2014	II	EOC or PPC	ramucirumab 8 mg/kg Q2W	60	14	2	-
Zhu *et al.*	2013	II	HCC	ramucirumab 8 mg/kg Q2W	42	0	3	-

mCRC: metastatic colorectal cancer, GAC: gastric cancer, GEJC: gastroesophageal junction cancer, NSCLC: non small cell lung cancer, MBC: metastatic breast cancer, RCC: renal cell carcinoma, EOC: epithelial ovarian carcinoma, PPC: primary peritoneal carcinoma, HCC: hepatocellular carcinoma, PTX: paclitaxel, DOC: docetaxel, PEM: pemetrexed, PLT: platinum, ERI: eribulin, DAC: dacarbazine.

### Quality of the studies

The Jadad scores of the included five muticenter, randomized, placebo controlled and double-blinded phase III trials ranged from 3 to 5, while there were two phase II, randomized, open-labeled studies were assigned a Jadad score of 2 and 3, respectively. Therefore, quality of these studies was fair and acceptable.

### Incidence of all-grade hemorrhagic events

**Figure 2:**
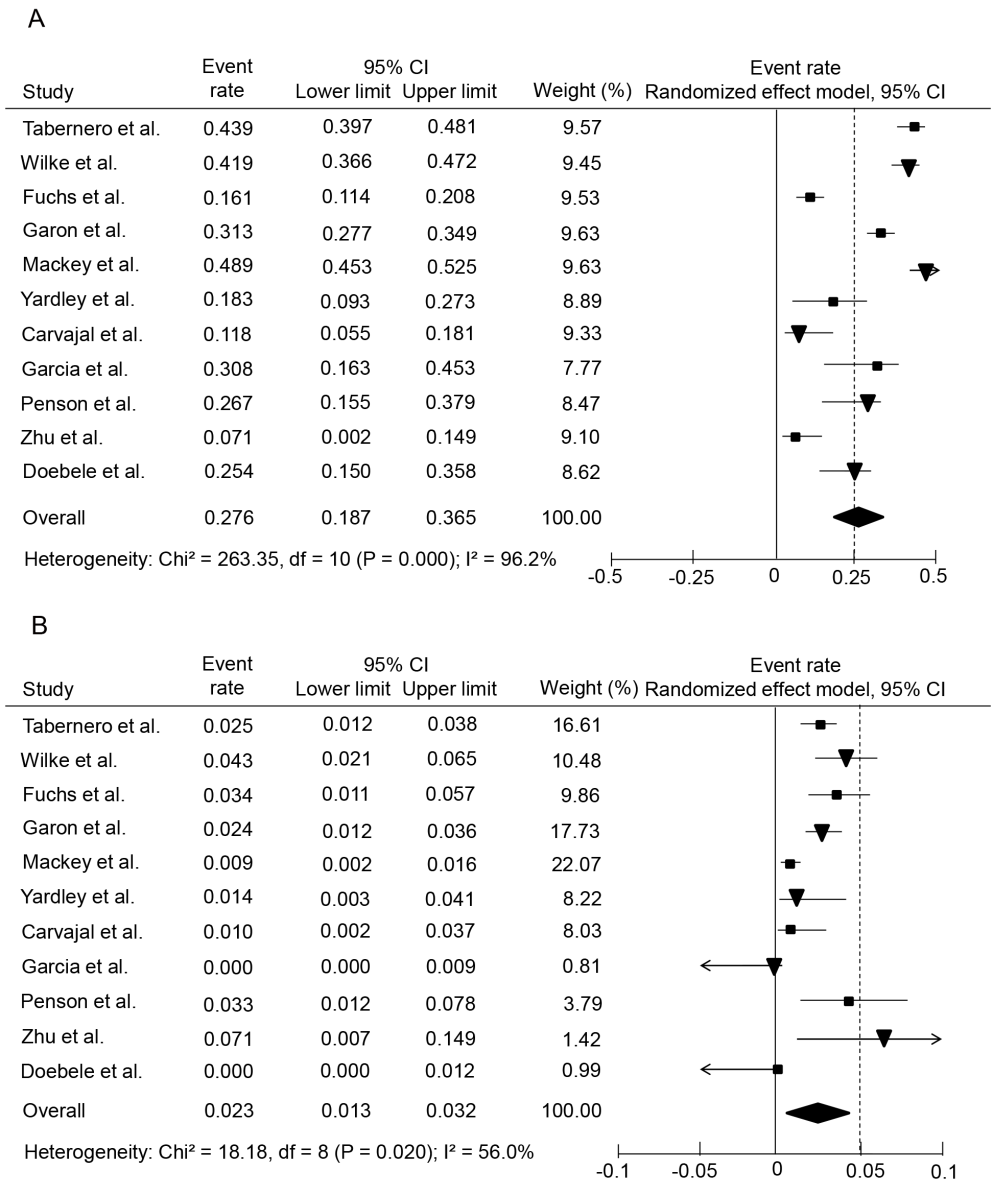
Forest plots of overall incidences of all-grade and high-grade hemorrhagic events in cancer patients treated with ramucirumab **A.**, overall incidence of all-grade hemorrhagic events; **B.**, overall incidence of high-grade hemorrhagic events.

For the incidence analysis, we considered arms receiving either ramucirumab monotherapy or ramucirumab-based combination. Thus, data for all-grade hemorrhagic events with ramucirumab was available for 4963 patients from all of the included trials [4-8, 10-15]. The incidence of all-grade hemorrhagic events ranged between 7.1 and 48.9%, with the lowest incidence seen in a phase II single arm trial among patients with HCC [15] and the highest incidence observed in a trial of patients with MBC [8]. Our meta-analysis showed a significant heterogeneity among included studies (*I^2^* = 96.2%, *p* = 0.000), and the calculated overall incidence of all-grade hemorrhagic events among cancer patients receiving ramucirumab was 27.6% (95% CI, 18.7-36.5%) using a random effects model (Figure 2A).

### Incidence of high-grade hemorrhagic events

The incidence of high-grade hemorrhagic events ranged between 0 to 7.1%, with the highest incidence observed in a phase II trial conducted by Zhu et al. in patients with HCC [19], and the lowest incidence noted in patients with RCC and NSCLC [13, 10]. Using a random effects model, the calculated overall incidence of high-grade hemorrhagic events among patients receiving ramucirumab was 2.3% (95% CI, 1.3-3.2%) (*I^2^* = 56.2% *p* = 0.02) (Figure 2B).

### Relative risks of low-grade and high-grade hemorrhagic events

The hemorrhagic events associated with ramucirumab might be related to several potential risk factors such as tumor type, the use of chemotherapeutic agents or other factors. In order to define the specific contribution of ramucirumab to the development of hemorrhagic events, and to exclude the potential impact of any confounding factors, we thus calculated the overall RR of hemorrhagic events from those randomised clinical trials in which ramucirumab was compared to controls in cancer patients who received concurrent chemotherapy or placebo.

**Figure 3:**
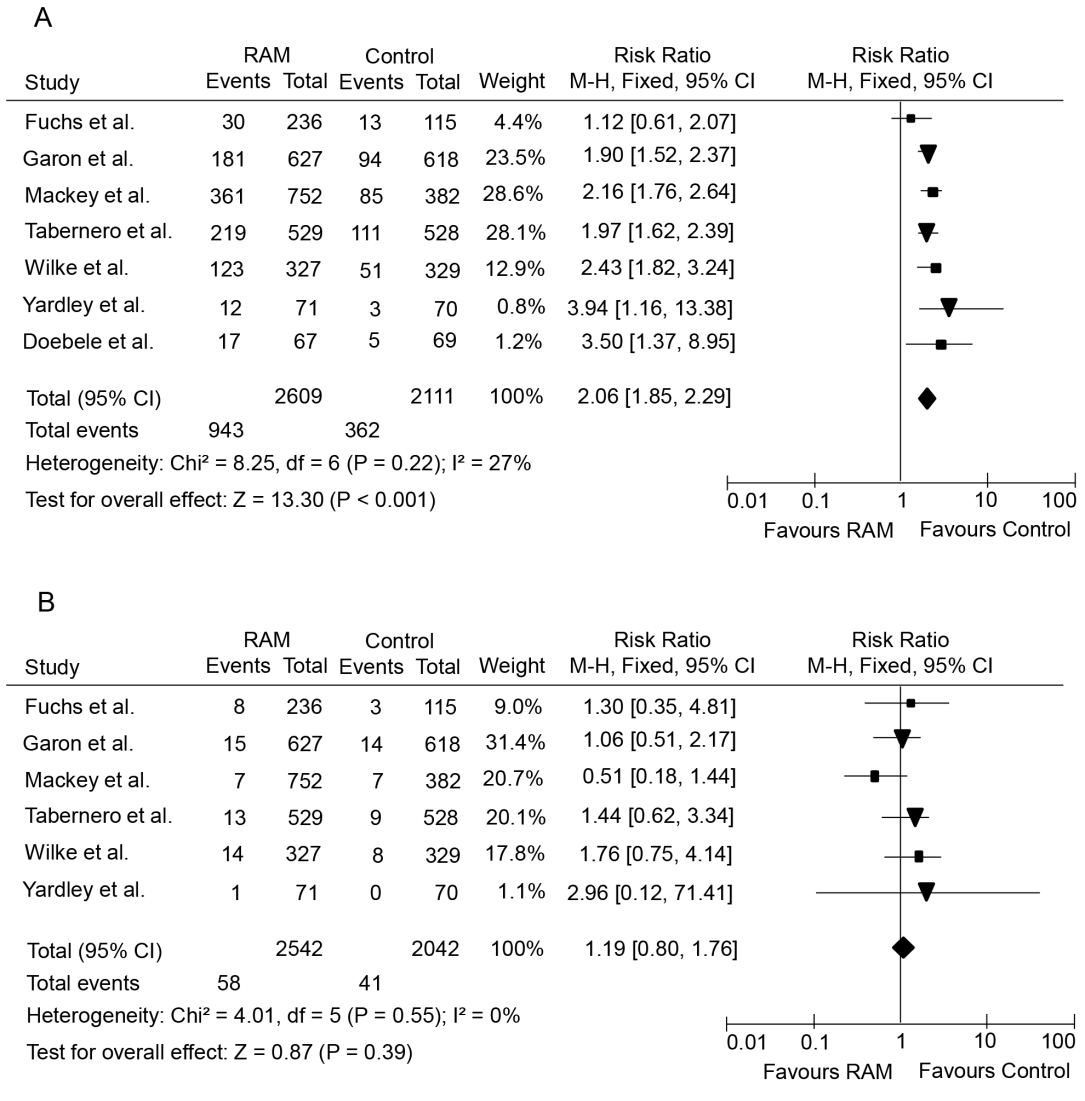
Forest plots of relative risks of low-grade and high-grade hemorrhagic events in cancer patients treated with ramucirumab *versus* control **A.**, relative risk of low-grade hemorrhagic events; **B.**, relative risk of high-grade hemorrhagic events.

Altogether, seven RCTs were pooled [4-8, 10, 11], including five phase III and two phase II studies. The pooled analysis showed that the administration of ramucirumab significantly increased the risk of developing all-grade hemorrhagic events in cancer patients with a RR of 2.06 (95% CI, 1.85-2.29; *p* < 0.001) using a fixed effects model (*I^2^* = 27%, *p* = 0.22) (Figure 3A). However, the RR for high-grade hemorrhagic events was comparable between ramucirumab containing and control treatment arms (RR = 1.19; 95% CI, 0.80-1.76, *p* = 0.39) with a fixed effects model (*I^2^* = 0.00%, *p* = 0.55) (Figure 3B).

### Relative risks of hemorrhagic events according to different tumor types

Patients with different malignancies might be at different risks of developing hemorrhagic events, mainly due to the different contributions from tumor biological behavior and associated treatment strategy. We thus examined if patients who are with a specific type of tumor have higher risk of developing hemorrhagic events compared with other cancers. As demonstrated in Table 2, the risks of developing low-grade hemorrhagic events were comparable between patients with different malignancies (the RRs for GAC or GEJC, NSCLC and MBC were 2.02, 95% CI, 0.72-5.67; 2.37, 95% CI, 1.81-3.10 and 3.28, 95% CI, 2.49-4.31, respectively). Whereas high-grade hemorrhagic events were more frequently observed in patients with GAC or GEJC (RR, 1.63; 95% CI, 0.78-3.42).

**Table 2:** Relative risk of hemorrhagic events associated with ramucirumab among patients with different tumor types and dosage regimens

	Number of	Relative risk (95% CI)	
	studies	Low-grade	High-grade
Overall	7	2.06 (1.85-2.29)^*^	1.19 (0.80-1.76)^#^
GAC or GEJC	2	2.02 (0.72-5.67)	1.63 (0.78-3.42)
NSCLC	2	2.37 (1.81-3.10)	NE
MBC	2	3.28 (2.49-4.31)	0.63 (0.24-1.68)
Ramucirumab 8mg/kg Q2W	3	2.02 (1.73-2.36)	1.54 (0.89-2.65)
Ramucirumab 10mg/kg Q3W	4	2.10 (1.81-2.43)	0.88 (0.50-1.56)

NE, not evaluable;

* for seven randomized controlled trials (RCTs) with record of low-grade hemorrhagic events;

# for six RCTs with toxicity profiles on high-grade hemorrhagic events.

### Relative risks of hemorrhagic events according to different ramucirumab dosage regimens

To understand further the role of ramucirumab in the pathogenesis of hemorrhagic events, we assessed whether the dosage regimen of ramucirumab was related to the risk of hemorrhagic events. Thus, the overall RRs of hemorrhagic events with regimen A (8mg/kg Q2W) and regimen B (10mg/kg Q3W) of ramucirumab were determined. As demonstrated in Table 2, the risks of developing low-grade hemorrhagic events were comparable between patients with regimen A (RR, 2.02; 95% CI, 1.73-2.36) and regimen B (RR, 2.10; 95% CI, 1.81-2.43). Whereas the RR of high-grade hemorrhagic events with regimen A (RR, 1.54; 95% CI, 0.89-2.65) was higher than that of regimen B (RR, 0.88; 95% CI, 0.50-1.56).

### Publication bias

**Figure 4:**
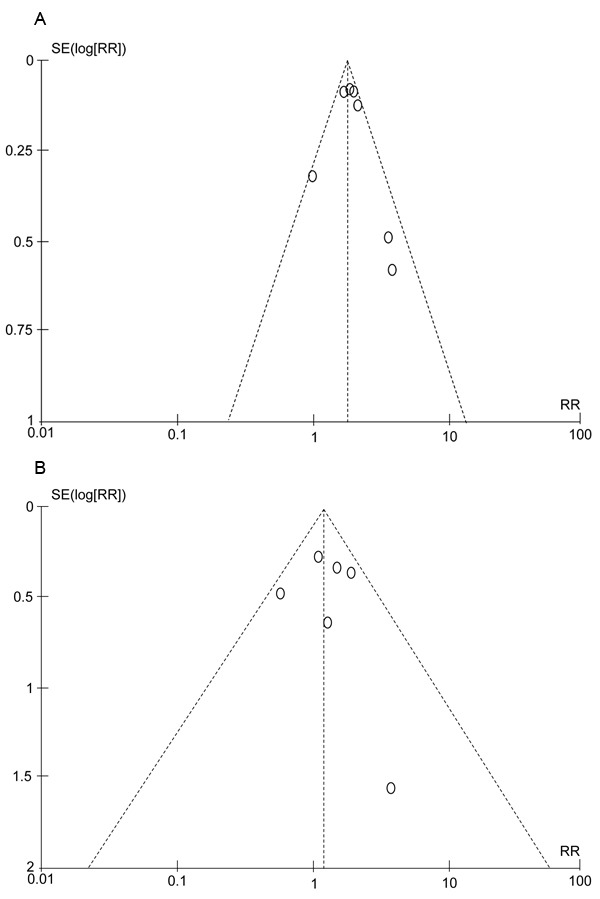
Funnel plots for relative risks of included studies in the meta-analysis **A.**, low-grade hemorrhagic events in cancer patients treated with ramucirumab; **B.**, high-grade hemorrhagic events in cancer patients treated with ramucirumab.

The potential publication biases were determined by performing funnel plots. And the funnel plots did not show any evidence of obvious asymmetry or publication bias (Figure 4).

## DISCUSSION

VEGF/VEGFR is extensively involved in vascular endothelial cell proliferation and endothelial cell survival, as well as in maintaining vascular integrity. By blocking VEGF/VEGFR pathway, antiangiogenic inhibitors not only inhibits tumor growth, but also impairs the repair capacity of endothelial cells and causes defects of the plasma membrane or underlying matrix, thus resulting to an increased risk of hemorrhage/bleeding [16]. In addition, life-threatening hemorrhage/bleeding may occur through weakening of the wall of major vessels by tumor erosion, necrosis or other concurrent pathological conditions [17-18]. Similar to other antiangiogenic inhibitors, hemorrhagic events (epistaxis, gastrointestinal hemorrhage/bleeding and pulmonary hemorrhage) is one of the major adverse events in cancer patients who received ramucirumab, and the reported incidences vary substantially among different clinical trials. The aim of this study was to define the overall incidence and relative risk of hemorrhagic events in cancer patients treated with ramucirumab. The present meta-analysis combined 11 publications including seven randomized controlled trials. Our meta-analysis demonstrated that ramucirumab was associated with an increased risk of developing hemorrhagic events, especially grade 1 and 2 hemorrhagic events. The overall incidence of all-grade and high-grade hemorrhagic events was 27.6% (95% CI, 18.7-36.5%) and 2.3% (95% CI, 1.3-3.2%), respectively. And our results from RCTs demonstrated that the relative risk of hemorrhagic events of ramucirumab compared to control was significantly increased for all-grade (RR, 1.97; 95% CI, 1.79-2.18, *p* < 0.001), but not for high-grade (RR, 1.19, 95% CI, 0.80-1.76, *p* = 0.36) hemorrhagic events.

Our study also demonstrated that the RRs of hemorrhagic events with ramucirumab varied depending on different tumor types and ramucirumab dosages. The results revealed that the RRs of developing low-grade hemorrhagic events were comparable among patients with different malignancies and ramucirumab dosages. Whereas a higher risk of high-grade hemorrhagic events was more frequently observed in patients with GAC or GEJC and those who received ramucirumab at a dose of 8mg/kg Q2W. The underlying mechanism might mainly be related to differences in tumor biological behavior, the location of the tumors, invasion of the gastrointestinal tract, tumor necrosis [19].

Administration of ramucirumab is associated with an increased risk of hemorrhagic events (including severe and sometimes life-threatening bleeding episodes), which is emphasized by a black-box warning in the prescribing information, suggesting monitoring patients for signs and symptoms of severe bleeding. For patients who are with high-grade hemorrhagic events, ramucirumab should not be administered.

The following limitations must be taken into account when interpreting the results of our meta-analysis. The main limitation was that some of the studies included into our analysis were RCTs while the others were not. As a result, the quality of the evidence could not be guaranteed. Besides, due to the inadequate random sequence generation and blinding procedure, it tended to increase the risk of bias of this meta-analysis. In addition, our meta-analysis was based on published results, but not individual patient data. Finally, although no efforts were spared to search the literature comprehensively, there still existed the possibility that few relevant publications were not identified.

In conclusion, our results indicate that the administration of ramucirumab in cancer patients is associated with an increased risk of low-grade, but not high-grade hemorrhagic events, which are modest and manageable while patients could continue to receive ramucizumab treatment to achieve their maximum clinical benefits.

## References

[1] Kerbel RS (2008). Tumor angiogenesis. N Engl J Med.

[2] Ferrara N (2004). Vascular endothelial growth factor: basic science and clinical progress. Endocr Rev.

[3] Spratlin JL, Cohen RB, Eadens M, Gore L, Camidge DR, Diab S, Leong S, O'Bryant C, Chow LQ, Serkova NJ, Meropol NJ, Lewis NL, Chiorean EG (2010). Phase I pharmacologic and biologic study of ramucirumab (IMC-1121B), a fully human immunoglobulin G1 monoclonal antibody targeting the vascular endothelial growth factor receptor-2. J Clin Oncol.

[4] Tabernero J, Yoshino T, Cohn AL, Obermannova R, Bodoky G, Garcia-Carbonero R, Ciuleanu TE, Portnoy DC, Van Cutsem E, Grothey A, Prausová J, Garcia-Alfonso P, Yamazaki K (2015). Ramucirumab *versus* placebo in combination with secondline FOLFIRI in patients with metastatic colorectal carcinoma that progressed during or after first-line therapy with bevacizumab, oxaliplatin, and a fluoropyrimidine (RAISE): a randomised, double-blind, multicentre, phase 3 study. Lancet Oncol.

[5] Wilke H, Muro K, Van Cutsem E, Oh SC, Bodoky G, Shimada Y, Hironaka S, Sugimoto N, Lipatov O, Kim TY, Cunningham D, Rougier P, Komatsu Y (2014). Ramucirumab plus paclitaxel *versus* placebo plus paclitaxel in patients with previously treated advanced gastric or gastro-oesophageal junction adenocarcinoma (RAINBOW): a double-blind, randomised phase 3 trial. Lancet Oncol.

[6] Fuchs CS, Tomasek J, Yong CJ, Dumitru F, Passalacqua R, Goswami C, Safran H, dos Santos LV, Aprile G, Ferry DR, Melichar B, Tehfe M, Topuzov E (2014). Ramucirumab monotherapy for previously treated advanced gastric or gastro-oesophageal junction adenocarcinoma (REGARD): an international, randomised, multicenter, placebo-controlled, phase 3 trial. Lancet.

[7] Garon EB, Ciuleanu T-E, Arrieta O, Prabhash K, Syrigos KN, Goksel T, Park K, Gorbunova V, Kowalyszyn RD, Pikiel J, Czyzewicz G, Orlov SV, Lewanski CR (2014). Ramucirumab plus docetaxel *versus* placebo plus docetaxel for second-line treatment of stage IV non-small-cell lung cancer after disease progression on platinum-based therapy (REVEL): a multicentre, double-blind, randomised phase 3 trial. Lancet.

[8] Mackey JR, Ramos-Vazquez M, Lipatov O, McCarthy N, Krasnozhon D, Semiglazov V, Manikhas A, Gelmon KA, Konecny GE, Webster M, Hegg R, Verma S, Gorbunova V (2014). Primary results of ROSE/TRIO-12, a randomized placebo-controlled phase III trial evaluating the addition of ramucirumab to first-line docetaxel chemotherapy in metastatic breast cancer. J Clin Oncol.

[9] Poole RM, Vaidya A (2014). Ramucirumab: first global approval. Drugs.

[10] Doebele RC, Spigel D, Tehfe M, Thomas S, Reck M, Verma S, Eakle J, Bustin F, Goldschmidt J, Cao D, Alexandris E, Yurasov S, Camidge DR (2015). Phase 2, randomized, open-label study of ramucirumab in combination with first-line pemetrexed and platinum chemotherapy in patients with ,nonsquamous, advanced/metastatic non-small cell lung cancer. Cancer.

[11] Yardley DA, Richards PD, Reeves JA, Dees EC, Osborne CR.C., Soliman HH, Paul D, Ademuyiwa FO, Guthrie TH, Bromund JL, Xu YH, Ibrahim AB (2014). Final results of a phase 2 study of ramucirumab (RAM) plus eribulin (E) *versus* E in advanced metastatic breast cancer (MBC). J Clin Oncol.

[12] Carvajal RD, Wong MK, Thompson JA, Gordon MS, Lewis KD, Pavlick AC, Wolchok JD, Rojas PB, Schwartz JD, Bedikian AY (2014). A phase 2 randomised study of ramucirumab (IMC-1121B) with or without dacarbazine in patients with metastatic melanoma. Eur J Cancer.

[13] Garcia JA, Hudes GR, Choueiri TK, Stadler WM, Wood LS, Gurtler J, Bhatia S, Joshi A, Hozak RR, Xu Y, Schwartz JD, Thompson JA (2014). A phase 2, single-arm study of ramucirumab in patients with metastatic renal cell carcinoma with disease progression on or intolerance to tyrosine kinase inhibitor therapy. Cancer.

[14] Penson RT, Moore KM, Fleming GF, Braly P, Schimp V, Nguyen H, Matulonis UA, Banerjee S, Haluska P, Gore M, Bodurka DC, Hozak RR, Joshi A (2014). A phase II study of ramucirumab (IMC-1121B) in the treatment of persistent or recurrent epithelial ovarian, fallopian tube or primary peritoneal carcinoma. Gynecol Oncol.

[15] Zhu AX, Finn RS, Mulcahy M, Gurtler J, Sun W, Schwartz JD, Dalal RP, Joshi A, Hozak RR, Xu Y, Ancukiewicz M, Jain RK, Nugent FW (2013). A phase II and biomarker study of ramucirumab, a human monoclonal antibody targeting the VEGF receptor-2, as first-line monotherapy in patients with advanced hepatocellular cancer. Clin Cancer Res.

[16] Kilickap S, Abali H, Celik I (2003). Bevacizumab, bleeding, thrombosis, and warfarin. J Clin Oncol.

[17] Kamba T, McDonald DM (2007). Mechanisms of adverse effects of anti-VEGF therapy for cancer. Br J Cancer.

[18] Peng L, Bu ZB, Zhou Y, Ye X, Liu J, Zhao Q (2014). Hemorrhagic events in cancer patients treated with aflibercept: a meta-analysis. Tumor Biol.

[19] Hapani S, Chu D, Wu SH (2009). Risk of gastrointestinal perforation in patients with cancer treated with bevacizumab: a meta-analysis. Lancet Oncol.

